# Sociocultural Factors Influencing Human *Streptococcus suis* Disease in Southeast Asia

**DOI:** 10.3390/foods11091190

**Published:** 2022-04-20

**Authors:** Anusak Kerdsin, Mariela Segura, Nahuel Fittipaldi, Marcelo Gottschalk

**Affiliations:** 1 Faculty of Public Health, Kasetsart University, Chalermphrakiat Sakon Nakhon Province Campus, Sakon Nakhon 47000, Thailand; 2 Research Group on Infectious Diseases in Production Animals (GREMIP) and Swine and Poultry Infectious Diseases Research Center (CRIPA), Faculty of Veterinary Medicine, University of Montreal, St-Hyacinthe, QC J2S 2M2, Canada; mariela.segura@umontreal.ca (M.S.); n.fittipaldi@umontreal.ca (N.F.); marcelo.gottschalk@umontreal.ca (M.G.)

**Keywords:** *Streptococcus suis*, Southeast Asia, sociocultural, raw pork dish, serotype, sequence type

## Abstract

The public health systems of Southeast Asian countries are financially challenged by a comparatively higher incidence of human *S. suis* infections than other geographical areas. Efforts to improve practices in production settings, including improved meat inspection regulations, prevention of the slaughtering of non-healthy pigs, and enhanced hygiene practices at processing facilities, along with improvements in the pork supply chain, all appear promising for reducing food cross-contamination with *S. suis*. However, opportunities for intervention at the societal level are also needed to effect changes, as population behaviors such as the consumption of raw pork, blood, and offal products are important contributors to the increased incidence of human *S. suis* disease in Southeast Asia. A plethora of factors are associated with the consumption of these high-risk dishes, including traditional culture and knowledge, shared beliefs, socio-economic level, and personal attitudes associated with gender and/or marital status. Education and intervention in behavioral attitudes that are sensible to cultural practices and traditions may provide additional means to reduce the burden of *S. suis* human disease in Southeast Asia.

## 1. Introduction

In recent years, the number of reported human *Streptococcus suis* cases has substantially increased, with Southeast Asian countries leading the counts [1,2]. Traditionally regarded in industrialized countries as an occupational disease affecting workers in close contact with infected pigs or contaminated pork-derived products, the emergence of human *S. suis* disease in Southeast Asia has been linked to foodborne infections [1]. This important zoonotic pathogen is classified into 29 serotypes. Serotype 2 is the most frequently recovered from human infections, although human cases due to serotypes 4, 5, 7, 9, 14, 16, 21, 24, and 31 have been reported [2,3,4,5,6,7,8,9,10].

In countries such as the United Kingdom, Spain, Germany, the Netherlands, Canada, and the United States (henceforth referred to as Western countries), as well as in Japan, China, and Hong Kong, most human *S. suis* cases have occurred after occupational exposure involving pig handling among pig farmers, bleeders, abattoir workers, carcass cutting and processing workers, butchers, and cooks [11,12]. As discussed in this review, while individuals working in the porcine industry are also at risk, reports from Thailand and Vietnam have shown that the proportion of patients with occupational exposure is lower than in Western countries. Indeed, in these non-Western countries, a non-trivial number of human cases have occurred in individuals consuming meals containing raw pork meat, blood, and other related products [7,13,14,15]. Thus, the available data clearly support the notion that consumption of these foods plays an important role in *S. suis* transmission, dissemination, and infection in Southeast Asia. In this review, we focus on the societal and cultural characteristics and behaviors, regional customs, and the individual and collective attitudes associated with high-risk food consumption leading to *S. suis* human disease.

## 2. Human *S. suis* Serotypes and Sequence Types in Southeast Asia

As summarized in Table 1, knowledge of serotypes and sequence types (STs) causing human *S. suis* disease has mostly been generated in Thailand and Vietnam. Human *S. suis* isolates from Southeast Asia are more diverse in terms of both the serotypes and STs present than those in Europe and the Americas. This may be due to the higher incidence of human *S. suis* disease in Southeast Asia in comparison to Western countries.

Reported serotypes of *S. suis* from humans in Southeast Asia are 2, 4, 5, 9, 14, 16, 24, and 31 [3,4,5,6,7,9,16,17]. Some of these STs, such as ST101-ST104, ST106, ST107, ST126, ST127, ST159-ST161, ST181, ST221, ST232-ST237, ST325, ST326, ST379-ST382, ST391-ST395, ST512-ST516, ST869, ST951, ST1656, ST1687, and ST1688 have so far exclusively been found in Southeast Asia (https://pubmlst.org/organisms/streptococcus-suis, accessed on 15 December 2021). The overall serotype and ST diversity among human *S. suis* isolates is higher in Thailand than in other countries in Southeast Asia. However, in Vietnam, CC1 has more STs than Thailand and other countries. Some STs (1, 105, 144) of serotypes 2 or 14 were found in both Thailand and Vietnam, which may indicate a higher circulation of these strains in this region.

## 3. Burden and Impact of *S. suis* Infection in Southeast Asia

The increasing number of human infections due to *S. suis* in Southeast Asian countries has had a major economic impact on public health systems. A study from Thailand showed that *S. suis* human infections in that country are responsible for an estimated loss in productivity-adjusted life years to the gross domestic product of USD 11.3 million, which equates to USD 36,033 lost per person [26]. A study in Chiang Mai (Northern Thailand) showed that the health burden measured in term of disability-adjusted life years (DALY) was estimated at 7.41 per 100,000 population [27]. Years of life lost (YLL) due to *S. suis* were higher in males (49 YYL) than in females (16 YYL). The economic impact of *S. suis* in Chiang Mai between 2013 and 2014 was, on average, THB 37,955 (GBP 759 or USD 1018) per patient. Out-of-pocket expenses for individuals and their families averaged THB 5198 (GBP 104 or USD 140) per patient [27]. In addition, another study revealed that in Vietnam, the direct cost per *S. suis* infection episode was USD 1635, consisting of USD 1046 and 589 for medical and non-medical costs, respectively [28], and that for the period 2011–2014, the annual direct cost was approximately USD 370,000–500,000. Importantly, the Vietnamese study reported that the indirect cost was substantially higher, reaching up to USD 2,270,000–2,880,000 and the DALY lost were in the range 1401–1866 for the period 2011–2014 [28].

Hearing loss and balance system dysfunction are the most frequently reported sequelae of patients who survive *S. suis* infections, which aggravate the impact of this disease on health and the quality of life of affected persons. Patients with long-term severe hearing loss paid significantly higher medical costs (USD 765) than those with non-severe impairment (USD 505) [28]. Another study from Vietnam showed significantly higher problems with mobility, self-care, performance of usual activities, and emotional impact caused by hearing impairment and dizziness [29]. Disability due to profound or complete hearing loss with vestibular dysfunction caused a burden of 397–516 DALY during 2011–2014 [28].

Another study revealed a high cost per patient associated with an *S. suis* serotype 2 outbreak [30]. That study investigated a large outbreak in Chiang Mai, Thailand in June–July 2008 (the second largest outbreak in Thailand). The outbreak had 32 confirmed cases, 2 probable cases, and 30 suspected cases. Per patient expenses were THB 38,370 (USD 1193). Outbreak-associated expenses are not a regular cost for hospital or public health routine functions and were mainly due to increases in antibiotics usage, medical supplies, laboratory investigations, outbreak investigations, and patient care related to patient treatment and measures taken to control the outbreak.

## 4. Contamination of *S. suis* in the Pork Supply Chain

The rise in the demand for pork and pork products has led to an increased risk of exposure to this zoonotic pathogen through contact with or consumption of meat from farms and market chains, which can impact national public health systems. High pig densities and frequent consumption of raw or undercooked pork are related to over 50% of the total human *S. suis* cases in Asia [31]. Understanding pig production and slaughtering and consumption practices or behaviors are critical in identifying or minimizing the risks of *S. suis* exposure to those working closely with pigs or pork meat, or to those consuming raw pig products.

Asymptomatic pigs, non-clinically affected at the time of slaughter, have been suggested as the primary source of *S. suis* introduced to slaughterhouses [32,33,34,35]. It is unknown whether some of these animals were ill just before being sent to the abattoir. Evidence from Vietnam and Thailand demonstrated that *S. suis* strains isolated from slaughterhouse pigs have pulse-field gel electrophoresis patterns and sequence types identical or highly related to those from diseased pigs and human isolates [33,36]. A study from Thailand revealed that the risk factors for contamination of *S. suis* were significantly higher in non-registered [OR (odds ratio) = 9.62, 95% CI (confidence interval) = 2.20–41.91; *p* value < 0.02] than in registered slaughterhouses [37]. Lack of proper identification of symptomatic and highly infective animals prior to slaughter and poor meat inspection are risk factors for the transmission of this zoonosis. In addition, access to protective equipment when handling raw pork products at slaughter points or kitchens is still limited in many rural areas in several Southeast Asian countries [31,38]. Therefore, improper slaughtering can result in *S. suis* contamination of pork meat, thus increasing the risk of infection to workers and consumers.

A study from Thailand showed that *S. suis* can contaminate the environment throughout the pig supply chain, from slaughterhouses up to retail markets [39]. Contamination of *S. suis* was reported on the working surfaces at slaughtering sites (20.8%), workers’ hands (16.7%), boots (16.7%), and pork meat (0.93%) [38]. Additionally, *S. suis* was isolated from surface swabs in pig transport trucks and from surface swabs of retail markets tables, cutting boards (but not knives), scales, and fridges [39]. Several studies from Thailand showed contamination of pork meat with *S. suis* [40,41]. Improper hygienic practices during carcass and pork handling may be responsible, at least in part, for the cross-contamination of carcasses, the environment, and raw pork product dishes with *S. suis*.

## 5. Sociocultural Aspects of Human *Streptococcus suis* Disease in Southeast Asian Countries

Ethnicity, culture, religion, and regional customs also play an important role in influencing the type of food eaten and its preparation in several Southeast Asian countries. A summary of traditional dishes of raw or undercooked pork, blood, and offal products frequently served in these countries is shown in Table 2.

### 5.1. Thailand

Although the annual incidence rates of human *S. suis* infections have not been consistently determined, two studies have estimated them [14,42]. A study conducted in Phayao province (Northern Thailand) in 2010 showed an incidence rate of *S. suis* infections in humans of 6.2 per 100,000 of the general population [14]. Given the incidence rate of this disease and the population in Northern Thailand, the number of human cases can be estimated at 730 per year in this region. The second study was conducted in Nakhon Phanom province (Northeastern Thailand) and found a much lower annual incidence of 0.1–2.2 cases per 100,000 population during 2006–2012 [42]. The reasons for these different rates are not known, but ethnicity origin, tribe, cultural behavior, and lifestyles might all influence the *S. suis* infection rates.

Four large outbreaks of *S. suis* infections in humans have been recorded in Thailand, mainly in Northern Thailand [2]. In a retrospective study, it was reported that between 2006 and 2012, the incidence of *S. suis* disease peaked during the rainy season (June–August) and that human cases appeared to have increased in accordance with rising rainfall [7,13]. A study from Vietnam showed a temporal and spatial association of occurrence of human *S. suis* meningitis cases with porcine reproductive and respiratory syndrome virus (PRRSv) outbreaks on pig farms [43]. In that study, human cases increased from April to July (highest peak in July) and from September to October (highest peak in October), these being periods that coincide with the rainy season in Thailand. A highly virulent PRRSv strain was introduced into Thai swine herds in early 2010 and caused major outbreaks [44]. Thus, an association between a PRRSv outbreaks and human *S. suis* cases in Thailand seems likely. However, more studies are needed to evaluate whether the introduction of PRRSv may have influenced the pattern of *S. suis* isolation from humans in Thailand.

In contrast, a prospective study in Phayao province, Thailand showed a peak incidence between April and May (summer), and most cases were related to the Songkran Festival (a traditional Thai New Year festival in Thailand) as well as other harvesting festivals during this period in this region [14]. Interestingly, consumption of the raw pork or blood dishes usually served during these events is common [45,46] http://www.pngo.moph.go.th/pngo/images/meeting2/620621/2106-5.pdf (accessed on 3 January 2022).

**Figure 1:**
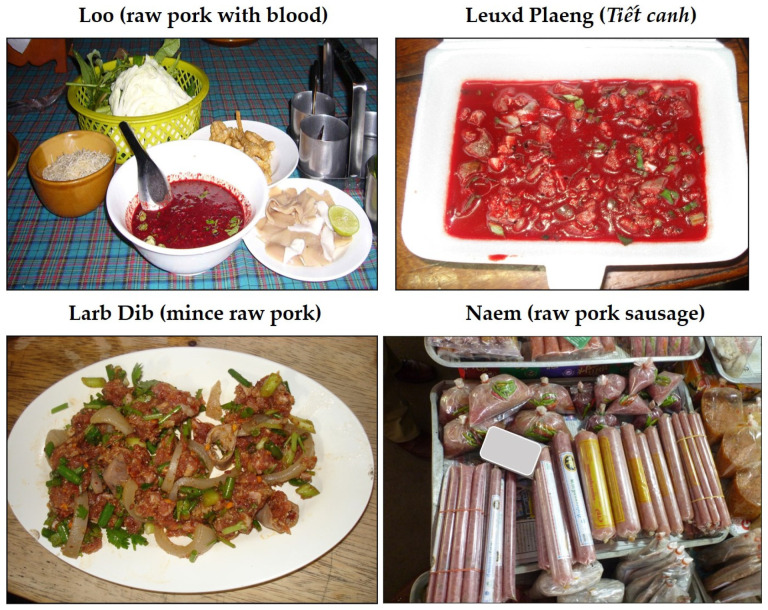
High-risk dishes available in Thailand.

The study conducted in Phayao province confirmed that more than 70% of cases with *S. suis* infections were associated with the consumption of raw pork and raw blood products, in Thai known as ‘*loo*’ [14]. Similarly, three large outbreaks were mainly linked to the consumption of raw pork and blood products, such as ‘*loo*’ and ‘*larb dib*’ (a spicy minced raw pork dish), in Thailand and ‘*leuxd plaeng*’ (or ‘*tiết canh*’—well-cooked pork with raw blood pudding, in Vietnam) (Figure 1). Some Thai people believe the raw pork/blood dish can promote health and that lemon and some herbs added to the dishes can inhibit or kill pathogens. These raw pork dishes are also commercially available at some traditional markets in this region and are sometimes shared in ritual ceremonies, such as Buddhist ordinations, weddings, housewarmings, and funerals [45,47,48].

Several studies conducted in Northern Thailand revealed that people, particularly in rural areas, have insufficient knowledge of the health risks posed by the consumption of raw pork or partially cooked pork products in certain ritual ceremonies [49,50,51]. On the other hand, a study in Chiang Mai revealed that although most people were aware that they should not eat uncooked meat, more than 95% of infected patients claimed that they, nonetheless, preferred to eat raw pork and blood dishes [27]. Most people who ate raw meat claimed they like its “delicious, sweet flavor”. They also tended to consume raw meat dishes with alcohol, which might lower their inhibitions [45]. Although risk communication activities have been and should continue to be implemented, this habit is a strong characteristic of the local culture and is practiced broadly across different age groups.

### 5.2. Vietnam

The estimated annual incidence rate of *S. suis* human disease between 2011 and 2014 ranged between 0.249 and 0.324 per 100,000 population [28]. Most of the bacterial meningitis cases in adults are due to *S. suis* infections [17,52]. Although the first prospective study of *S. suis* infection in humans in Southern Vietnam reported that 66.9% of the patients were not exposed to pigs or pork products, the possibility of consumption of undercooked pork and raw pig blood (which is also common in Vietnam) cannot be ruled out [17]. A study in Northern Vietnam revealed that only 16 out of 43 patients had been in close contact with pigs or pork products: 8 patients exposed to slaughtered pigs, 5 exposed to pork products, and 3 had consumed raw pig blood. The remaining ~62% of patients did not report contact with pigs or pork products [52]. Another study from Vietnam showed that 48% of patients with *S. suis* infections had eaten high-risk dishes (undercooked pig blood and pig intestine) during the 2 weeks prior to admission [53].

Consumption of raw pig blood dishes, or ‘*Tiết canh’*, is a common practice among Vietnamese people (Figure 1). The dish is widely served during traditional family cerebrations, such as weddings. A survey study conducted in rural and urban areas reported that rural residency, gender, age, occupation, low-income urban residents, and married people in rural areas were associated with consumption of raw pork and pig blood dishes [15]. As in Thailand, adult working-age males are more inclined to eat raw pork and pig blood than are females. Additionally, there is a widespread belief among the Vietnamese that eating ‘*tiết canh*’ leads to health benefits, such as preventing anemia, or has a general cooling effect. Only a low proportion of people knew the specific diseases transmissible to humans through consumption of this dish [15]. Active education on behavioral attitudes regarding the health risk posed by consuming raw pork or partially cooked pork products may help effect a reduction in human *S. suis* disease.

### 5.3. Lao People’s Democratic Republic (Lao PDR)

No prevalence data are available for *S. suis* infections in Lao PDR. However, human *S. suis* cases have been documented in the country [1]. In Lao PDR, pigs are an important source of food and income, being raised by many rural residents. Proximity with livestock poses a risk of zoonotic infection via direct contact, environmental contamination, or consumption of unsafe meat products, such as raw or undercooked pork, raw pig’s blood, and fermented pork sausage [38]. As is the case in both Thailand and Vietnam, consumption of raw meat dishes is also a common practice in parts of the country where the *S. suis* disease has been reported [31].

Several traditional dishes in Lao PDR use raw meat either in the form of flesh or specific organs or blood as their main ingredient (*larb dib*) [54]. Tran et al. (2006) reported that raw meat consumption was practiced by about 30% of parasite-infected patients (*Taenia solium*) in central Lao PDR, and Xayaseng and colleagues (2013) reported that all focus group participants consumed dishes with raw fish due to the visual appearance, taste, smell, and personal preferences [55,56]. A cluster of angiostrongyliasis cases involved the consumption of a raw or undercooked meal of wild *Monitor* lizard [54]. Although no report has confirmed it yet in Lao PDR, this behavior may be relevant for human *S. suis* disease. The important cross-border trade between Lao PDR and Vietnam, the shared sociocultural beliefs, and the propensity for raw meat consumption by the Lao people, taken together, indicate a real risk for the emergence of zoonotic diseases, including human *S. suis* disease.

### 5.4. Indonesia

No data on the prevalence of *S. suis* infections are yet available. However, 44 human *S. suis* meningitis cases were reported in Bali between 2014 and 2017. Although no epidemiological data of the risk factors related to these meningitis cases are available, eating raw meat with fresh blood, a common practice in that region, was suspected to be the route for *S. suis* transmission [57,58]. Another study from 2020 showed human *S. suis* meningitis male cases related to consumption of raw pork during Mebat, an important ceremonial tradition of Balinese Hindu people. In that case, two of the male’s friends who participated in the Mebat and ate the raw pork dish also experienced the same symptoms as the patient [59]. The Mebat tradition serves the meat in dishes named red ‘*lawar*’ and ‘*komoh*’ soup. Red ‘*lawar*’ is prepared from minced pork mixed with raw pork blood and vegetables, while ‘*komoh*’ soup is prepared using fresh pork blood and Balinese herbs [59]. The consumption of raw pork or undercooked pork products is a strong risk factor for the transmission of *S. suis* infection, as described above.

### 5.5. Other Countries in Southeast Asia

Traditional consumption of raw pork product dishes in Malaysia, Brunei, Singapore, the Philippines, Myanmar, Cambodia, and Timor-Leste has not been properly documented. However, two different reports of human *S. suis* disease in the USA described people returning from traveling in the Philippines who had a history of ingestion of raw or partially cooked pork products while previously in the country [19,20]. Human *S. suis* infection cases have been related to occupational infections in Malaysia [21]. In Cambodia, no human *S. suis* infection has been linked to a history of raw pork consumption or to the oral route of infection [22,56]. In Singapore, a human *S. suis* case related to occupational exposure was reported in 1997, but other reported cases in the country had no history of either pig or pork contact or of consumption of raw or partially cooked pork products [23,24,25].

## 6. Risk Assessment of Food Safety and Consumption of Raw Pork Dishes

A meta-analysis of risk factors for *S. suis* infection in 850 human disease cases among 3 case-control studies was conducted in Thailand by comparison with community controls and diagnosed non-*S. suis* sepsis cases. The study revealed that major risk factors for human *S. suis* disease were raw pork consumption (OR = 78), exposure to pigs or pork (OR = 3.03), pig-related occupations (OR = 3.07), and male gender (OR = 5.84) [60]. A different study from Vietnam showed that risk factors for human *S. suis* infections were eating high-risk dishes (OR 4.44), pig-related occupations (OR 5.52), pig exposure while having skin injuries (OR 15.96), and male gender (OR 3.53) [53]. An outbreak investigation in Phayao province in Thailand in 2007 showed that crude ORs of ingesting blood, intestines and other internal organs in raw pork dishes were 48.0, 17.5, and 10.1, respectively. However, the statistically most significant risk factor was eating blood, with an adjusted OR of 24.8, based on multivariate analysis [45].

An additional study by Wongnak and colleagues (2020) evaluated the *S. suis* serotype 2 illness risk from consuming pork in Thailand using quantitative microbial risk assessment (QMRA) [39]. The QMRA model revealed a baseline of the daily probability of *S. suis* serotype 2 illness corresponding to 502 annual cases and 31 deaths. In a scenario where 80% of retail pork is contaminated with viable *S. suis,* the estimated risk increased to 56.8 times the baseline value, with 28,525 cases and 1757 fatal cases [39]. This study showed that a reduction in the initial bacterial concentration by 0.5 log CFU/g would decrease the risk by about 50%. The reduction of the undercooked pork consumption rate to 0.1% contributed up to 30 times fewer expected annual cases [39].

Takeuchi and colleagues (2017) reported that the implementation of a food safety campaign in Phayao province in Thailand during 2011–2013 led to a marked decrease in the annualized incidence of *S. suis* human disease, from 6.4/100,000 people in 2010 (before implementation) to 2.7/100,000 people in 2011, then to 2.0/100,000 persons in 2012, and finally to 3.5/100,000 persons in 2013 [61]. Overall, there was a 3.94/100,000 persons decrease in incidence after the campaign (*p* < 0.001). This campaign success was linked to activities such as knowledge transfer to residents through the public health network; in particular, face-to-face education with small groups seemed to be effective. However, this study also showed a small increase in the number of cases in 2013. Therefore, while behavioral changes following a 1-year food safety campaign led to a significant improvement, a continuous campaign would be preferable to sustain those gains over even longer periods. Some have suggested that introducing educational programs in childhood or adolescence may be effective for further behavioral change [61].

## 7. Policy Implications

In Vietnam, national *S. suis* guidelines on the clinical and laboratory diagnosis, treatment, and prevention of *S. suis* have been issued to all hospitals [62]. The guidelines contained a description of the clinical syndrome and the organism, recommendations on microbiological diagnosis, and a recommendation to treat suspected cases with ampicillin (a third-generation cephalosporin) and intravenous corticosteroids [62]. A scientific partnership between an international research group and an influential national institute closely linked to government was key for instrumenting policy change at the national level [62].

In Thailand, the Ministry of Public Health regulates the protocols and establishes the surveillance and rapid response team (SRRT). When outbreaks or disease are found either in a hospital or a community, a SRRT team goes to investigate and reports back to the hospital and to the Ministry’s department of disease control. All SRRT activities, processes, and evidence, including the outcome, must be measured and referred back to headquarters within 72 h [63]. National guidelines for human *S. suis* infections are not yet available. However, a practice of *S. suis* recruitment in the public health system is followed according to the above description. The *S. suis* cases from the hospitals have been reported into the R506 system (a daily case report of communicable diseases) of the Ministry of Public Health after the first large outbreak on 2007. A manual of *S. suis* outbreak investigation and a specific investigation form were provided to the provincial public health offices (SRRT headquarters) when outbreaks occurred.

Both Thailand and Vietnam have a similar policy of tertiary care or treatment. However, *S. suis* health literacy and education, hygiene, and food-safety practices have not been tackled consistently by national policies. Policy makers could also instrument change by increasing efforts on surveillance reports and epidemiological surveys at the community level.

## 8. Conclusions and Perspectives

Human *S. suis* disease in Southeast Asia is unique in that, in addition to occupational risks, cultural, religious, and other societal behaviors and attitudes associated with food consumption play an important role. Raw meat consumption has been associated with: (1) predisposing factors, including male gender, marital status, primary school education, and knowledge and beliefs; (2) enabling factors, including meat accessibility, meat with low prices, absence of health information via several social media, or absence of community rules; and (3) reinforcing factors, including family members and friends who share a predilection for eating raw meat dishes [64]. Thus, effecting a reduction in the burden of human *S. suis* disease in Southeast Asia will require a concerted effort by governments and communities. Some actions, such as improved meat inspection regulations and hygiene practices at pork processing facilities, align directly within government and public health mandates and can be enforced. Governments can also establish educational programs for preventing this infection and to reduce its morbidity and mortality. Campaigns involving education on factors such as the links between healthy animals and healthy food, and active education programs related to general food safety, including the mandatory use of personal protective equipment during slaughtering pigs and processing pork into dishes, are some strategies that can be used by officials and administrators.

Changing consumption practices is also as an important avenue to reduce human *S. suis* disease in Southeast Asia. However, it may be very difficult to effect changes rapidly given the many cultural and societal factors involved. Top-down approaches, such as government bans of high-risk dishes may be unenforceable and ineffective, not only because of strong traditions and practices but also because of the financial implications. Indeed, there is a well-established industry around these dishes, which is fueled by consumer demand. The industry, as well as consumers, may simply reject bans. Multilevel approaches that respect the essence of these traditions and practices and that actively engage community, religious, and other social leaders may offer an opportunity to implement the needed changes in behavioral patterns. Preventative measures should be implemented with the support of the community, whether related to education programs or to risk communication at primary health care centers in the area. Culturally sensible educational approaches targeting high-risk groups such as adult men or alcohol drinkers offer the most promise, but early education intervention from primary school may provide broader, long-term gains, as knowledge is gained and behaviors and attitudes are taught to the next generations. School-led sanitation programs in other developing countries have shown success in effecting changes [38].

In addition to production setting regulations, hygiene, behavioral changes, health education, and medical or diagnostic practices can also play a role. Early *S. suis* identification in sick patients seeking care using rapid alternative methods rather than traditional culture (the gold standard) could facilitate prompt treatment and reduce the mortality rate. Several alternative methods have been developed and directly applied to clinical specimens of *S. suis*-infected patients including PCR, real-time PCR, whole-genome sequencing, and colloidal gold–based immunochromatographic strip testing [65,66,67,68]. Interestingly, a study developed a screening questionnaire tool for *S. suis* infection among patients that was composed of the history of raw pork or blood consumption, exposure or handling of pork or pig products, alcoholic beverage consumption, and age over 25 years [69]. It had 90.2% accuracy, 92.3% reliability, 92.3% sensitivity, 50% specificity, 92.3% positive-predictive value, and 25% negative-predictive value [69]. The specificity increased if the patients had signs and symptoms such as fever, headache, myalgia, nausea or vomiting, a stiff neck, or hearing loss [69]. This tool could be useful in endemic areas, especially in the countries of Southeast Asia.

Thus, reducing the burden of *S. suis* disease in Southeast Asia requires a multidimensional approach combining government and public health efforts through regulations and education, active community involvement to effect behavioral changes that are evidence-based but culturally sensible and acceptable, and the adoption by healthcare systems of more rapid diagnostics and more relevant screening tools.

## Figures and Tables

**Table 1:** Serotypes and sequence types of human *S. suis* reported in Southeast Asia.

Country	Serotype	Clonal Complex	Sequence Type	Reference
Thailand	2	1	1, 11, 105, 126, 144, 298, 337	[4,5,6,7,9,16], https://pubmlst.org/organisms/streptococcus-suis (accessed on 3 January 2022)
		25	25, 102, 103, 380, 381, 395, 515, 516	
		28	28, 382	
		104	101, 104, 391, 392, 393, 512, 513, 514	
		233/379	233, 379, 1656	
		1687/1688	1687, 1688	
		Singleton	236	
	4	94	94	
	5	221/234	221	
		Singleton	181, 235	
	9	16	16	
	14	1	11, 105, 127	
	24	221/234	221, 234	
	31	221/234	221	
Vietnam	2	1	1, 105, 107, 144, 159, 160, 161, 325, 326, 869, 951	[3,17], https://pubmlst.org/organisms/streptococcus-suis (accessed on 3 January 2022)
	14	1	1, 105	
	16		106	
Indonesia	2 or 1/2	ND	ND	[18]
The Philippines	ND	ND	ND	[19,20]
Malaysia	ND	ND	ND	[21]
Cambodia	2	ND	ND	[22]
Singapore	ND	ND	ND	[23,24,25]
Laos	ND	ND	ND	[1]

ND = No data.

**Table 2:** High-risk traditional dishes commonly served in some Southeast Asian countries.

Country	High-Risk Dishes	Description	Related to Human *S. suis* Infections
Thailand	*Loo*	Raw pig’s blood soup combined with a spice mixture and served with raw pig’s kidney, raw pork, crispy deep-fried noodles, and kaffir lime leaf.	Yes
	*Larb Dib*	Minced raw pork mixed with seasonings, roasted rice, and vegetables. Sometimes, blood may be mixed with the minced raw pork.	Yes
	*Leuxd Plaeng* or *Tiết Canh*	Raw blood pudding mixed with cooked pork and offal products, together with crushed peanuts and chopped herbs	Yes
	*Naem* (synonym: *som moo*, *naem maw*, *chin som*)	A red-colored, semi-dry, fermented minced raw pork and pork skin sausage.	No report yet
Vietnam	*Tiết Canh*	Raw blood pudding mixed with cooked meat such as pork and offal products, together with crushed peanuts and chopped herbs.	Yes
Lao PDR	*Larb Dib*	Minced raw pork mixed with seasonings, roasted rice, and vegetables.	No report yet
Indonesia	*Lawar*	Minced pork mixed with raw pig’s blood and vegetables.	Yes
	*Komoh*	Fresh pig’s blood and Balinese herbs soup.	Yes

## Data Availability

Not applicable.

## References

[1] Goyette-Desjardins G., Auger J.P., Xu J., Segura M., Gottschalk M. (2014). *Streptococcus suis*, an important pig pathogen and emerging zoonotic agent—An update on the worldwide distribution based on serotyping and sequence typing. Emerg. Microbes Infect..

[2] Segura M., Aragon V., Brockmeier S.L., Gebhart C., Greeff A., Kerdsin A., O’Dea M.A., Okura M., Saléry M., Schultsz C. (2020). Update on *Streptococcus suis* Research and Prevention in the Era of Antimicrobial Restriction: 4th International Workshop on *S. suis*. Pathogens.

[3] Nghia H.D., Hoa N.T., Linh L., Campbell J., Diep T.S., Chau N.V., Mai N.T., Hien T.T., Spratt B., Farrar J. (2008). Human case of *Streptococcus suis* serotype 16 infection. Emerg. Infect. Dis..

[4] Kerdsin A., Oishi K., Sripakdee S., Boonkerd N., Polwichai P., Nakamura S., Uchida R., Sawanpanyalert P., Dejsirilert S. (2009). Clonal dissemination of human isolates of *Streptococcus suis* serotype 14 in Thailand. J. Med. Microbiol..

[5] Kerdsin A., Dejsirilert S., Sawanpanyalert P., Boonnark A., Noithachang W., Sriyakum D., Simkum S., Chokngam S., Gottschalk M., Akeda Y. (2011). Sepsis and spontaneous bacterial peritonitis in Thailand. Lancet.

[6] Kerdsin A., Hatrongjit R., Gottschalk M., Takeuchi D., Hamada S., Akeda Y., Oishi K. (2017). Emergence of *Streptococcus suis* serotype 9 infection in humans. J. Microbiol. Immunol. Infect..

[7] Kerdsin A., Akeda Y., Takeuchi D., Dejsirilert S., Gottschalk M., Oishi K. (2018). Genotypic diversity of *Streptococcus suis* strains isolated from humans in Thailand. Eur. J. Clin. Microbiol. Infect. Dis..

[8] Callejo R., Prieto M., Salamone F., Auger J.P., Goyette-Desjardins G., Gottschalk M. (2014). Atypical *Streptococcus suis* in man, Argentina, 2013. Emerg. Infect. Dis..

[9] Hatrongjit R., Kerdsin A., Gottschalk M., Takeuchi D., Hamada S., Oishi K., Akeda Y. (2015). First human case report of sepsis due to infection with *Streptococcus suis* serotype 31 in Thailand. BMC Infect. Dis..

[10] Liang P., Wang M., Gottschalk M., Vela A.I., Estrada A.A., Wang J., Du P., Luo M., Zheng H., Wu Z. (2021). Genomic and pathogenic investigations of *Streptococcus suis* serotype 7 population derived from a human patient and pigs. Emerg. Microbes Infect..

[11] Gottschalk M., Xu J., Calzas C., Segura M. (2010). *Streptococcus suis*: A new emerging or an old neglected zoonotic pathogen?. Future Microbiol..

[12] Dutkiewicz J., Sroka J., Zając V., Wasiński B., Cisak E., Sawczyn A., Kloc A., Wójcik-Fatla A. (2017). *Streptococcus suis*: A re-emerging pathogen associated with occupational exposure to pigs or pork products. Part I—Epidemiology. Ann. Agric. Environ. Med..

[13] Kerdsin A., Dejsirilert S., Puangpatra P., Sripakdee S., Chumla K., Boonkerd N., Polwichai P., Tanimura S., Takeuchi D., Nakayama T. (2011). Genotypic profile of *Streptococcus suis* serotype 2 and clinical features of infection in humans, Thailand. Emerg. Infect. Dis..

[14] Takeuchi D., Kerdsin A., Pienpringam A., Loetthong P., Samerchea S., Luangsuk P., Khamisara K., Wongwan N., Areeratana P., Chiranairadul P. (2012). Population-based study of *Streptococcus suis* infection in humans in Phayao Province in northern Thailand. PLoS ONE.

[15] Huong V.T., Hoa N.T., Horby P., Bryant J.E., Van Kinh N., Toan T.K., Wertheim H.F. (2014). Raw pig blood consumption and potential risk for *Streptococcus suis* infection, Vietnam. Emerg. Infect. Dis..

[16] Takamatsu D., Wongsawan K., Osaki M., Nishino H., Ishiji T., Tharavichitkul P., Khantawa B., Fongcom A., Takai S., Sekizaki T. (2008). *Streptococcus suis* in humans, Thailand. Emerg. Infect. Dis..

[17] Mai N.T., Hoa N.T., Nga T.V., Linh L., Chau T.T., Sinh D.X., Phu N.H., Chuong L.V., Diep T.S., Campbell J. (2008). *Streptococcus suis* meningitis in adults in Vietnam. Clin. Infect. Dis..

[18] Susilawathi N.M., Tarini N., Fatmawati N., Mayura P., Suryapraba A., Subrata M., Sudewi A., Mahardika G.N. (2019). *Streptococcus suis*-associated meningitis, Bali, Indonesia, 2014–2017. Emerg. Infect. Dis..

[19] Wongjittraporn S., Teerasukjinda O., Yee M., Chung H.H. (2014). *Streptococcus suis* meningoencephalitis with seizure from raw pork ingestion: A case report. Hawaii J. Med. Public Health.

[20] Lee G.T., Chiu C.Y., Haller B.L., Denn P.M., Hall C.S., Gerberding J.L. (2008). *Streptococcus suis* meningitis, United States. Emerg. Infect. Dis..

[21] Rajahram G.S., Hameed A.A., Menon J., William T., Tambyah P.A., Yeo T.W. (2017). Case report: Two human *Streptococcus suis* infections in Borneo, Sabah, Malaysia. BMC Infect. Dis..

[22] Vlieghe E. (2014). The Microbiological Spectrum of Invasive Bacterial Infections in Cambodian Adults and Implications for Standard Treatment Guidelines. Master’s Thesis.

[23] Tambyah P.A., Kumarasinghe G., Chan H.L., Lee K.O. (1997). *Streptococcus suis* infection complicated by purpura fulminans and rhabdomyolysis: Case report and review. Clin. Infect. Dis..

[24] Chan Y.C., Wilder-Smith A., Ong B.K., Kumarasinghe G., Wilder-Smith E. (2002). Adult community acquired bacterial meningitis in a Singaporean teaching hospital. A seven-year overview (1993–2000). Singap. Med. J..

[25] Tan J.H., Yeh B.I., Seet C.S. (2010). Deafness due to haemorrhagic labyrinthitis and a review of relapses in *Streptococcus suis* meningitis. Singap. Med. J..

[26] Rayanakorn A., Ademi Z., Liew D., Lee L.H. (2021). Burden of disease and productivity impact of *Streptococcus suis* infection in Thailand. PLoS Negl. Trop. Dis..

[27] Thongsawad S. (2016). Burden and Epidemiological Characterisations of *Streptococcus suis* in Chiang Mai, Thailand. Ph.D. Thesis.

[28] Huong V., Turner H.C., Kinh N.V., Thai P.Q., Hoa N.T., Horby P., van Doorn H.R., Wertheim H. (2019). Burden of disease and economic impact of human *Streptococcus suis* infection in Viet Nam. Trans. R. Soc. Trop. Med. Hyg..

[29] Huong V., Long H.B., Kinh N.V., Ngan T., Dung V., Nadjm B., van Doorn H.R., Hoa N.T., Horby P., Wertheim H. (2018). Long-term outcomes of patients with *Streptococcus suis* infection in Viet Nam: A case-control study. J. Infect..

[30] Wongsawat S. (2010). *Streptococcus suis* serotype 2 outbreak at Chomthong district, Chiang Mai province, June–July, 2008. Lanna Public Health J..

[31] Burniston S., Okello A.L., Khamlome B., Inthavong P., Gilbert J., Blacksell S.D., Allen J., Welburn S.C. (2015). Cultural drivers and health-seeking behaviours that impact on the transmission of pig-associated zoonoses in Lao People’s Democratic Republic. Infect. Dis. Poverty.

[32] Meekhanon N., Kaewmongkol S., Phimpraphai W., Okura M., Osaki M., Sekizaki T., Takamatsu D. (2017). Potentially hazardous *Streptococcus suis* strains latent in asymptomatic pigs in a major swine production area of Thailand. J. Med. Microbiol..

[33] Ngo T.H., Tran T.B., Tran T.T., Nguyen V.D., Campbell J., Pham H.A., Huynh H.T., Nguyen V.V., Bryant J.E., Tran T.H. (2011). Slaughterhouse pigs are a major reservoir of *Streptococcus suis* serotype 2 capable of causing human infection in southern Vietnam. PLoS ONE.

[34] Nutravong T., Angkititrakul S., Jiwakanon N., Wongchanthong W., Dejsirilerts S., Nawa Y. (2014). Identification of major *Streptococcus suis* serotypes 2, 7, 8 and 9 isolated from pigs and humans in upper northeastern Thailand. Southeast Asian J. Trop. Med. Public Health.

[35] Thongkamkoon P., Kiatyingangsulee T., Gottschalk M. (2017). Serotypes of *Streptococcus suis* isolated from healthy pigs in Phayao Province, Thailand. BMC Res. Notes.

[36] Kerdsin A., Takeuchi D., Nuangmek A., Akeda Y., Gottschalk M., Oishi K. (2020). Genotypic comparison between *Streptococcus suis* isolated from pigs and humans in Thailand. Pathogens.

[37] Prasertsang T., Cheveerach P. (2019). Risk factors on contamination of *Streptococcus suis* in slaughterhouse in Maha Sarakham province. KKU Vet. J..

[38] Holt H.R., Inthavong P., Khamlome B., Blaszak K., Keokamphe C., Somoulay V., Phongmany A., Durr P.A., Graham K., Allen J. (2016). Endemicity of zoonotic diseases in pigs and humans in Lowland and Upland Lao PDR: Identification of socio-cultural risk factors. PLoS Negl. Trop. Dis..

[39] Wongnak P., Wiratsudakul A., Nuanualsuwan S. (2020). A risk assessment of pathogenic *Streptococcus suis* in pork supply chains and markets in Thailand. Food Control.

[40] Boonyong N., Kaewmongkol S., Khunbutsri D., Satchasataporn K., Meekhanon N. (2019). Contamination of *Streptococcus suis* in pork and edible pig organs in central Thailand. Vet. World.

[41] Noppon B., Khaeng S., Sopa A., Phuaram P., Wongsan R., Laohasinnurak T. (2014). *Streptococcus suis* serotype 2 in uncooked pork meat products in Khon Kaen, northeastern Thailand, and their antimicrobial profiles. Int. J. Sci. Eng. Res..

[42] Praphasiri P., Owusu J.T., Thammathitiwat S., Ditsungnoen D., Boonmongkon P., Sangwichian O., Prasert K., Srihapanya S., Sornwong K., Kerdsin A. (2015). *Streptococcus suis* infection in hospitalized patients, Nakhon Phanom Province, Thailand. Emerg. Infect. Dis..

[43] Huong V.T., Thanh L.V., Phu V.D., Trinh D.T., Inui K., Tung N., Oanh N.T., Trung N.V., Hoa N.T., Bryant J.E. (2016). Temporal and spatial association of *Streptococcus suis* infection in humans and porcine reproductive and respiratory syndrome outbreaks in pigs in northern Vietnam. Epidemiol. Infect..

[44] Nilubol D., Tripipat T., Hoonsuwan T., Kortheerakul K. (2012). Porcine reproductive and respiratory syndrome virus, Thailand, 2010–2011. Emerg. Infect. Dis..

[45] Khadthasrima N., Hannwong T., Thammawitjaya P., Pingsusean D., Akkanij B., Jaikhar A., Paungmali P., Yudee P., Wongyai S., Samerchea S. (2008). Human *Streptococcus suis* outbreak in Phayao Province, Thailand, 2007. OSIR.

[46] Juntasiriyarkorn S., Henpraserttae N., Iamsirithaworn S., Thepsittha K., Saengrueng S. (2010). Outbreak verification summary-17th Week 25 April–1 May 2010. WESR.

[47] Fongcom A., Pruksakorn S., Netsirisawan P., Pongprasert R., Onsibud P. (2009). *Streptococcus suis* infection: A prospective study in northern Thailand. Southeast Asian J. Trop. Med. Public Health.

[48] Navacharoen N., Chantharochavong V., Hanprasertpong C., Kangsanarak J., Lekagul S. (2009). Hearing and vestibular loss in *Streptococcus suis* infection from swine and traditional raw pork exposure in northern Thailand. J. Laryngol. Otol..

[49] Kaewmoon P. (2009). Knowledge and Practice in Prevention of *Streptococcus suis* Infection among People in Tha-It Subdistrict, Mueang District, Uttaradit Province. Master’s Thesis.

[50] Yana A. (2008). Knowledge and Practice in Prevention of *Streptococcus suis* Infection among People in Mae-Narua Sub-District, Mueang District, Phayao Province. Master’s Thesis.

[51] Sutthaluang N., Korwanich K., Korwanich N. (2021). Perception and preventive behaviors for *Streptococcus suis* infection of people in Pua sub-district, Pua district, Nan province. Dis. Control J..

[52] Wertheim H.F., Nguyen H.N., Taylor W., Lien T.T., Ngo H.T., Nguyen T.Q., Nguyen B.N., Nguyen H.H., Nguyen H.M., Nguyen C.T. (2009). *Streptococcus suis*, an important cause of adult bacterial meningitis in northern Vietnam. PLoS ONE.

[53] Nghia H.D., Tu L., Wolbers M., Thai C.Q., Hoang N.V., Nga T.V., Thao L., Phu N.H., Chau T.T., Sinh D.X. (2011). Risk factors of *Streptococcus suis* infection in Vietnam. A case-control study. PLoS ONE.

[54] Yang L., Darasavath C., Chang K., Vilay V., Sengduangphachanh A., Adsamouth A., Vongsouvath M., Keolouangkhot V., Robinson M.T. (2021). Cluster of angiostrongyliasis cases following consumption of raw monitor lizard in the Lao People’s Democratic Republic and review of the literature. Trop. Med. Infect. Dis..

[55] Tran D.S., Odermatt P., Le T.O., Huc P., Druet-Cabanac M., Barennes H., Strobel M., Preux P.M. (2006). Prevalence of epilepsy in a rural district of central Lao PDR. Neuroepidemiology.

[56] Xayaseng V., Phongluxa K., van Eeuwijk P., Akkhavong K., Odermatt P. (2013). Raw fish consumption in liver fluke endemic areas in rural southern Laos. Acta Trop..

[57] Besung I., Suarjana I., Agustina K.K., Winaya I., Soeharsono H., Suwiti N.K., Mahardika G.N. (2019). Isolation and identification of *Streptococcus suis* from sick pigs in Bali, Indonesia. BMC Res. Notes.

[58] Aryasa I.A., Widiasari N.P.A., Susilawathi N.M., Fatmawati N.N.D., Adnyana I.M.O., Sudewi A.A.R., Adi Tarini N.M. (2020). *Streptococcus suis* meningitis related to processing and consuming raw pork during Balinese tradition, Mebat. Med. J. Indones.

[59] Rozo M., Schully K.L., Philipson C., Fitkariwala A., Nhim D., Som T., Sieng D., Huot B., Dul S., Gregory M.J. (2020). An observational study of sepsis in Takeo Province Cambodia: An in-depth examination of pathogens causing severe infections. PLoS Negl. Trop. Dis..

[60] Rayanakorn A., Goh B.H., Lee L.H., Khan T.M., Saokaew S. (2018). Risk factors for *Streptococcus suis* infection: A systematic review and meta-analysis. Sci. Rep..

[61] Takeuchi D., Kerdsin A., Akeda Y., Chiranairadul P., Loetthong P., Tanburawong N., Areeratana P., Puangmali P., Khamisara K., Pinyo W. (2017). Impact of a food safety campaign on *Streptococcus suis* infection in humans in Thailand. Am. J. Trop. Med. Hyg..

[62] Horby P., Wertheim H., Ha N.H., Trung N.V., Trinh D.T., Taylor W., Ha N.M., Lien T.T., Farrar J., Van Kinh N. (2010). Stimulating the development of national Streptococcus suis guidelines in Viet Nam through a strategic research partnership. Bull. World Health Organ..

[63] Soontornpipit P., Viwatwongkasem C., Taratep C., Teerawat W., Vanitchatchavan P. (2016). Development of the Electronic Surveillance Monitoring System on Web Applications. Procedia Comput. Sci..

[64] Sathawornwiwat A., Kengkanpanich M., Saengrat N., Sangian J. (2020). Factor predicting raw meat consumption behavior among people Chiang Klang District, Nan Province. Thail. J. Health Educ..

[65] Nga T.V., Nghia H.D., Tu L., Diep T.S., Mai N.T., Chau T.T., Sinh D.X., Phu N.H., Nga T.T., Chau N.V. (2011). Real-time PCR for detection of *Streptococcus suis* serotype 2 in cerebrospinal fluid of human patients with meningitis. Diagn. Microbiol. Infect. Dis..

[66] Nakayama T., Zhao J., Takeuchi D., Kerdsin A., Chiranairadul P., Areeratana P., Loetthong P., Pienpringam A., Akeda Y., Oishi K. (2014). Colloidal gold-based immunochromatographic strip test compromising optimised combinations of anti-*S. suis* capsular polysaccharide polyclonal antibodies for detection of *Streptococcus suis*. Biosens. Bioelectron..

[67] Zhang X., Wu Z., Wang K. (2020). Diagnosis of *Streptococcus suis* meningoencephalitis with metagenomic next-generation sequencing of the cerebrospinal fluid: A case report with literature review. BMC Infect. Dis..

[68] Thu I., Tragoolpua K., Intorasoot S., Anukool U., Khamnoi P., Kerdsin A., Tharinjaroen C.S. (2021). Direct detection of *Streptococcus suis* from cerebrospinal fluid, positive hemoculture, and simultaneous differentiation of serotypes 1, 1/2, 2, and 14 within single reaction. Pathogens.

[69] Thayawiwat C., Wichaikam O., Painpringam A. (2012). *Streptococcus suis* infection in the patients in Chiang Kham hospital Chiang Kham district, Phayao province 2009–2011: Screening test for *Streptococcus suis* infection. J. Prevent. Med. Assoc. Thail..

